# Decreased SFRP5 correlated with excessive metabolic inflammation in polycystic ovary syndrome could be reversed by metformin: implication of its role in dysregulated metabolism

**DOI:** 10.1186/s13048-021-00847-4

**Published:** 2021-07-20

**Authors:** Yi Zhang, Yuxin Ran, Lingna Kong, Lihong Geng, Hua Huang, Hongying Zhang, Jun Hu, Hongbo Qi, Ying Chen

**Affiliations:** 1 NHC Key Laboratory of Birth Defects and Reproductive Health, Chongqing Population and Family Planning Science and Technology Research Institute, Chongqing, People’s Republic of China; 2 Reproductive Medicine Center, Department of Obstetrics and Gynecology, The First Affiliated Hospital of Chongqing Medical University, Chongqing, People’s Republic of China; 3 School of Nursing, The First Affiliated Hospital of Chongqing Medical University, Chongqing, People’s Republic of China

**Keywords:** Polycystic ovary syndrome (PCOS), Secreted frizzled-related protein (SFRP) 5, Inflammation, Metformin, Hyperandrogenism (HA)

## Abstract

**Background:**

Polycystic Ovary Syndrome (PCOS) is a complex endocrine disorder of heterogeneous nature. Secreted frizzled-related protein (SFRP) 5 is an anti-inflammatory adipokine implicated in metabolic homeostasis. We aimed to confirm the correlation between SFRP5, metabolic inflammation and PCOS, investigate the predictive value of SFRP5 for PCOS and the involvement of SFRP5 in metformin treated PCOS.

**Methods:**

This retrospective case–control study included 140 PCOS and 33 control women. Sixty seven PCOS women were included for detecting serum SFRP5 level and its correlation with metabolic inflammation. Predictive value of SFRP5 for PCOS was evaluated by logistic regression and receiver operating characteristic (ROC) analyses. Seventy three PCOS women complicated with impaired glucose tolerance (IGT)/insulin resistance (IR) were included for investigating the effects of metformin (37 with metformin vs. 36 without metformin) on SFRP5, pro-inflammatory cytokines, ovulation and pregnancy rate.

**Results:**

Plasma SFRP5 levels were decreased in PCOS (odds ratio: 0.78, 95% confidence interval (CI):0.703–0.866, *P* < 0.001) independent of obesity. SFRP5 was negatively associated with IL-6, TNFα, FAI and HOMA-IR. The cut-off point of SFRP5 < 46.13 ng/ml was optimal to identify PCOS with a higher specificity of 96.87% and a relatively lower sensitivity compared to AMH. SFRP5 increased specificity of AMH for predicting PCOS, especially which with relatively decreased AMH (< 4.7 ng/ml). Metformin promoted SFRP5 and decreased leptin, IL-6 and TNFα secretion in PCOS women with metabolic abnormality in a time dependent manner and with improved ovulation rate and pregnancy rate.

**Conclusion:**

Decreased SFRP5 was associated with metabolic inflammation in PCOS and has a potential role for the supplement of AMH in predicting PCOS. The reverse of serum SFRP5 by metformin indicated that SFRP5 participated in the improvment of follicular development by metformin. Further prospective investigations are needed to confirm these preliminary data.

**Supplementary Information:**

The online version contains supplementary material available at 10.1186/s13048-021-00847-4.

## Background

Polycystic ovary syndrome (PCOS) is a complex endocrine disorder characterized by oligo-anovulation (OA), polycystic ovarian morphology (PCOM), hyperandrogenism (HA) and accompanied by metabolic aberrations (adverse lipid profile, impaired glucose tolerance (IGT), insulin resistance (IR) and type 2 diabetes mellitus (T2DM)). The prevalence of PCOS varies from 6 to 10% according to different diagnostic criteria, making PCOS the most common endocrine disorder among women of reproductive age [1].

The limited knowledge of its etiology and the heterogeneous nature make the definition, diagnostic criteria and treatment of PCOS to be controversial issues for many years [2, 3]. Approximately half of the PCOS women exhibit obesity. Besides, about 30 to 50% of lean PCOS women still have an increased risk of metabolic abnormalities [4, 5]. In addition, there are also a portion of PCOS women without abnormal glycolipid metabolism exhibiting HA and ovarian dysfunction. Although biomarkers such as anti-Müllerian hormone (AMH) have been suggested for diagnosis of PCOS, none of them could be used independently [6]. Appropriate recognition and management of PCOS remain challenges.

Increasing evidence supports that chronic low-grade inflammation correlats with HA and IR and emerges as a key contributor to the pathogenesis of PCOS [7]. Wnt signaling plays critical role in reproduction especially in the regulation of follicle maturation and is involved in the pathogenesis of PCOS [8, 9]. Secreted frizzled-related protein (SFRP) 5, a member of the Wnt inhibitor SFRP family, is recently identified as an anti-inflammatory adipokine implicated in metabolic homeostasis [10]. It is expressed in the mature adipocytes of mice and humans. SFRP5 not only strengthens adipocyte hypertrophy through repressing oxidative metabolism, but also modulates both inflammation and metabolic dysfunction depending on the type of tissue as well as its inflammatory and metabolic state [11]. SFRP5 correlates with susceptibility to obesity and is highly sensitive to the obesogenic environment. It has been reported decreased in a variety of inflammatory disorders such as obesity, type 2 diabetes mellitus (T2DM) and coronary heart disease (CHD) in multiple researches as an endogenous inhibitor of WNT5A signalling pathways [12–14]. However, controversial results were also found in other clinical studies [15]. SFRP5 expressed at similar levels in mural and cumulus granulosa cells of human and is the more abundant SFRP member in mural granulosa cells. SFRP5 expression was up-regulated by LH/hCG in luteinized human mural granulosa cells, which suggest SFRP5 might have a role in follicular development and ovulation [16]. However, the mechanism underlying is not clear. Metformin is extensively evaluated in the treatment of PCOS because of its insulin-lowering effect. Moreover, it can also relieve chronic low-grade inflammation, inhibit ovarian steroidogenesis (including androgen production), and increase ovulation rate of PCOS women [17–20]. However, few studies supported the relationship between SFRP5 and PCOS [21, 22]. The correlation of SFRP5 and metabolic inflammation in metformin treated PCOS women is unclear.

The purpose of this study was to confirm the correlation of SFRP5, metabolic inflammation and PCOS, investigate the predictive value of SFRP5 for PCOS. More importantly, we evaluated the effect of metformin on SFRP5, pro-inflammatory cytokines and ovulation rate in PCOS women with metabolic abnormalities to disclose the role of SFRP5 in metabolic inflammation of PCOS.

## Methods

### Study participants

This retrospective study included PCOS and control women of Chinese Han population visiting the reproductive medical center of the First Affiliated Hospital of Chongqing Medical University from January to July 2018. PCOS patients fulfilled the Rotterdam criteria containing at least two of the following three characteristics: (1) OA, (2) clinical and/or biochemical signs of HA, and (3) PCOM. Exclusion criteria were other etiologies for HA such as congenital adrenal hyperplasia, Cushing's syndrome, ovarian leydig cell tumour. Women with regular menstrual cycles were recruited as the controls. None of them had any known disease, were smokers or took any hormonal or other insulin-modifying therapy during the preceding two months.

The PCOS women were divided into two groups. One was gerenal PCOS included for descriptive research, which compared the differential serum level of SFRP5 in PCOS and its relationship with anthropometric and biochemical parameters. The PCOS women were also divided into subgroups according to the thresholds of body mass index (BMI) and AMH [6] indicated by previous studies: (1) BMI < 25 and BMI ≥ 25; (2) AMH < 4.7 ng/ml and AMH ≥ 4.7 ng/ml.

The other group of PCOS women was included for intervention research. They were complicated with IGT/IR and without other infertile factors and accepted no insulin-modifying therapy treatment in the previous six months were included for analyzing the role of SFRP5 in metformin treated PCOS. They were resistant to clomiphene in previous treatments and were divided into two groups. Group I was pretreated with metformin (1,000–1,500 mg/day; Conquer, Chongqing, China) for at least three months. Group II accepted no metformin treatment. Both groups were treated with clomiphene (150 mg/day for 5 consecutive days on menstrual cycle day 5; Codal-Synto, Limassol, and Cyprus). Human chorionic gonadotropin (hCG), 10,000 U (Livzon, Zhuhai, China) was administered intramuscularly whenever the diameter of the dominant follicle reached ≥ 18 mm and the estradiol level reached 150–200 pg/mL/follicle. Regular sexual intercourse was advised 24 h after the hCG day and afterward. If there were more than 3 dominant follicles, contraception was requested. The ovulation rates per cycle, days for follicular development, endometrial thickness, number of dominant follicles (> 14 mm), serum estradiol on the day of HCG, accumulated HCG positive rate, accumulated clinical pregnancy rate, miscarriage rate and multiple pregnancy rate were investigated.

### Anthropometric and biochemical measurements

Plasma glucose and hemoglobin A1c (HbA1c) levels were measured by the glucose-oxidase method and anion-exchange high-performance liquid chromatography, respectively. Total cholesterol, high-density lipoprotein cholesterol (HDL-C), triglycerides (TGs), and low-density lipoprotein cholesterol (LDL-C) were determined enzymatically using an auto analyzer (Hitachi, Tokyo, Japan). Serum insulin, total testosterone, follicle-stimulating hormone (FSH), luteinizing hormone (LH) and sex hormone-binding globulin (SHBG) levels were assayed by an automated chemiluminescence system on the UniCel Dxl 800 (Beckman Coulter, Fullerton, CA, USA) between day 2 and 5 of the menstrual cycle. Insulin resistance index (HOMA-IR) and Free androgen index (FAI) were calculated as following respectively: fasting glucose [mmol/L] * fasting insulin [IU/mL])/22.5 and testosterone (nmol/L) × 100/SHBG (nmol/L). The area under the curve of glucose (AUCG) and the area under the curve of insulin (AUCI) were calculated for detailed analysis of the OGTT (AUCG = (G0 + G3)/2 + G1 + G2 and AUCI = (I0 + I3)/2 + I1 + I2).

### Oral glucose tolerance test (OGTT)

The baseline blood samples were obtained after an overnight fasting of 12 h. Then blood samples were obtained at 0.5, 1 and 2 h for the measurements of glucose and insulin after dranking 75 g of glucose.

### Measurement of human SFRP5, TNFα, IL-6 and leptin serum concentrations

Fasting blood samples were analyzed for SFRP5, TNFα, IL-6 and leptin after 0, 1 and 3 months of metformin treatment in Group I using enzyme-linked immunosorbent assays (ELISAs) (USCN Life Science and CUSABIO, Wuhan, PR China) with a sensitivity of 0.60 ng/ml, 1.95 pg/ml, 1.95 pg/ml and 0.06 ng/ml, respectively.

### Statistical analysis

All statistical analyses were performed using SPSS 19.0 software (SPSS Inc., Chicago, IL, USA) and MedCalc 18.11.3 software (Acacialaan, Ostend, Belgium). Normally distributed continuous data are presented as the mean ± standard deviation (SD). Non-normally distributed data such as insulin and HOMA-IR are presented as medians (25th and 75th percentiles). Subjects with missing data were excluded. For normally distributed continuous data, comparisons among groups were performed by ANOVA and an unpaired *t* test as appropriate. For nonnormally distributed continuous data, we used Kruskal–Wallis ANOVA followed by pairwise comparisons or a Mann–Whitney U test to compare differences in the variables in normal controls and different PCOS subgroups and a Dunn-Bonferroni test for post hoc comparisons. Partial correlations between SFRP5 and other variables were calculated by controlling for the covariates. Simple and multivariable linear regression analyses examined the associations of SFRP5 with other variates. Stepwise logistic regression analyses were adjusting for age, BMI, AMH, AUCg, AUCins, HOMA-IR and FAI were conducted to assess the effects of SFRP5 in the diagnoses PCOS. The variables were log transformed to conform to normal distribution assumptions. The Box-Tidswell transformation (x*ln(x)) of the predictor x was used to assess the linearity in the logit. A receiver operating characteristic (ROC) analysis was performed to assess the diagnostic value of SFRP5 as a marker of PCOS. The maximum Youden index was used to determine the optimal sensitivity and specificity, as well as the corresponding cut-off value. *P* values of less than 0.05 were considered statistically significant.

## Results

### Circulating SFRP5 levels and its association with clinical and biochemical characteristics in PCOS

A total of 91 PCOS patients and 108 control women satisfied the inclusion criteria. However, only 67 PCOS and 33 control women with consent and available blood samples were enrolled. Thirty-one of PCOS were with PCOM, HA and OA, twenty-three with HA and OA and thirteen with PCOM and OA.

As displayed in Table 1, serum SFRP5 were significantly lower in PCOS women, *P* < 0.001. Instead, the levels of BMI, waist hip ratio (WHR), fasting blood glucose (FBG), fasting insulin (FINS), 2 h blood glucose and insulin, AUCG, AUCI, HOMA-IR, HbA1c, TG, AMH, FAI, IL-6 and TNFα(*P* < 0.001); TC (*P* = 0.007); HDL-C(*P* = 0.022) and LH (*P* = 0.023) were significantly higher in PCOS women.

**Table 1:** Comparison of main clinical features in normal controls and PCOS subjects

Item	Normal control	PCOS		*P-*value
		**Normal weight** **(BMI < 25)**	**Overweight /Obese** **(BMI ≥ 25)**	
**N**	**33**	**33**	**34**	
**SFRP5 (ng/ml)**	63.98 ± 12.40	37.99 ± 10.80^c^	32.77 ± 8.31^c^	< 0.001
**Age(years)**	27.97 ± 3.86	26.97 ± 2.82	29.06 ± 3.86	0.293
**BMI (Kg/m**^**2**^**)**	20.70 ± 2.14	21.62 ± 1.98	28.28 ± 2.40^c,f^	< 0.001
**WHR**	0.79 ± 0.03	0.79 ± 0.04	0.84 ± 0.02^c,f^	< 0.001
**AMH (ng/ml)**	2.86 ± 0.70	5.52 ± 2.02^c^	6.62 ± 2.83^c^	< 0.001
**FSH (mIU/ml)**	7.58 ± 1.95	6.18 ± 1.87^a^	6.89 ± 2.21	0.036
**LH (mIU/ml)**	7.01 ± 1.77	12.46 ± 9.99^a^	9.42 ± 6.24	0.023
**FAI**	2.15 ± 0.56	5.98 ± 2.47^c^	6.92 ± 3.09^c^	< 0.001
**FINS (Uiu/ml)**	5.4(4.3,6.5)	5.97(4.85, 9.15)	12.99(10.83,14.85)^c,f^	< 0.001
**2-h INS (Uiu/ml)**	18.5(13.22,27.87)	41.2 (33.94,81.95)^c^	99.49(67.99,138.86)^c,e^	< 0.001
**FBG (mmol/l)**	4.91 ± 0.23	5.31 ± 0.33^c^	5.71 ± 0.61^c^	< 0.001
**2-hBG (mmol/l)**	5.74 ± 1.00	7.58 ± 1.92^c^	9.01 ± 2.30^c^	< 0.001
**TG (mmol/L)**	1.00 ± 0.23	1.17 ± 0.58	2.27 ± 0.91^c,f^	< 0.001
**TC (mmol/L)**	4.36 ± 0.44	4.49 ± 0.51	4.78 ± 0.53^b^	0.007
**HDL-C (mmol/L)**	1.41 ± 0.31	1.28 ± 0.27	1.24 ± 0.22	0.022
**LDL-C (mmol/L)**	2.55 ± 0.40	2.55 ± 0.34	2.91 ± 0.41^b,e^	0.056
**HbA1c(%)**	5.35 ± 0.24	5.60 ± 0.28^b^	5.77 ± 0.28^c^	< 0.001
**AUCI**	75.66(66.10,88.95)	148.82(106.4,255.49)^c^	265.94(195.23,388.22)^c,e^	< 0.001
**AUCG**	18.70 ± 2.40	25.21 ± 4.87^c^	27.78 ± 4.69^c^	< 0.001
**HOMA-IR**	1.17(0.97,1.44)	1.54(1.01, 2.05)	3.28(2.56, 4.06)^c,f^	< 0.001
**IL6(pg/ml)**	4.48 ± 2.94	47.65 ± 30.68^c^	52.30 ± 39.06^c^	< 0.001
**TNFα (ng/ml)**	16.08 ± 3.17	61.36 ± 13.68^c^	61.27 ± 10.95^c^	< 0.001

Values are presented as mean ± standard deviation (SD) or medians (25th, 75th percentiles)

*WHR* waist-to-hip ratio, *AMH* anti-Müllerian hormone, *FAI* the free androgen index, *FBG* fasting blood glucose, *2-hBG* 2-h blood glucose, *FINS* fasting insulin, *2-h Ins* 2-h plasma insulin after glucose overload, *TG* triglyceride, *TC* total cholesterol, *HDL-C* high-density lipoprotein cholesterol, *LDL-C* low-density lipoprotein cholesterol, *AUCI* the area under the curve of insulin, *AUCG* the area under the curve of glucose, *HOMA-IR* the homeostasis model assessment of IR

*P* value: total PCOS compared to the control

a,b and c *p* < 0.05, *p* < 0.01 and *p* < 0.001 as compared to the control

d, e and f *p* < 0.05, *p* < 0.01 and *p* < 0.001 as compared to normal weight PCOS (BMI < 25)

No obvious difference of SFRP5 level was found between normal weight (BMI < 25) and overweight /obese (BMI ≥ 25) PCOS patients, although BMI, WHR, HOMA-IR and FINS levels were significantly higher in the latter. Furthermore, SFRP5 was still lower in normal-weight PCOS patients than BMI-matched control patients, *P* < 0.001.

After adjusting for age, BMI and WHR, partial correlations disclosed negative correlations of serum SFRP5 with FAI, FBG, IL-6 and TNFα (*P* < 0.001), AMH (*P* = 0.005), 2-h BG (*P* = 0.007) and HOMA-IR (*P* = 0.028) (Table 2). Multiple linear regression analysis suggested SFRP5 was only significantly correlated with FAI (*P* = 0.041), IL-6 (*P* = 0.046) and TNFα (*P* = 0.033) (Table 3).

**Table 2:** Partial correlations analysis of variables associated with circulating SFRP5 levels in study population

Item	Plasma SFRP5		Plasma SFRP5 (age-adjusted)		Plasma SFRP5 (age-BMI-adjusted)		Plasma SFRP5 (age-BMI-WHR-adjusted)	
	**r**	***P*-value**	**r**	***P*-value**	**r**	***P*-value**	**r**	***P*-value**
**Age(years)**	0.174	0.083						
**BMI (Kg/m**^**2**^**)**	-0.492	< 0.001	-0.524	< 0.001				
**WHR**	-0.271	0.006	-0.296	0.003	0.036	0.725		
**AMH (ng/ml)**	0.501	< 0.001	-0.498	< 0.001	-0.284	0.005	-0.285	0.005
**FAI**	-0.566	< 0.001	-0.560	< 0.001	-0.390	< 0.001	-0.400	< 0.001
**FINS (Uiu/ml)**	-0.452	< 0.001	-0.464	< 0.001	-0.158	0.121	-0.161	0.114
**2-h INS (Uiu/ml)**	-0.450	< 0.001	-0.459	< 0.001	-0.154	0.131	-0.161	0.115
**FBG (mmol/l)**	-0.510	< 0.001	-0.568	< 0.001	-0.400	< 0.001	-0.401	< 0.001
**2-hBG (mmol/l)**	-0.444	< 0.001	-0.484	< 0.001	-0.271	0.007	-0.274	0.007
**HOMA-IR**	-0.484	< 0.001	-0.505	< 0.001	-0.218	0.031	-0.223	0.028
**IL6(pg/ml)**	-0.550	< 0.001	-0.572	< 0.001	-0.463	< 0.001	-0.465	< 0.001
**TNFα (ng/ml)**	-0.708	< 0.001	-0.707	< 0.001	-0.601	< 0.001	-0.600	< 0.001

**Table 3:** Multiple linear regression analysis of variables associated with plasma SFRP5 levels in all subjects studied

Variable	Multiple	
	**Beta**	***P*-value**
**BMI**	-0.121	0.664
**AMH**	-0.013	0.241
**FAI**	-0.216	0.041
**FBG**	-0.112	0.206
**2-h BG**	0.007	0.940
**IL6**	-0.266	0.046
**TNFɑ**	-0.308	0.033

### The predictive value of SFRP5 for the diagnosis of PCOS

**Fig. 1:**
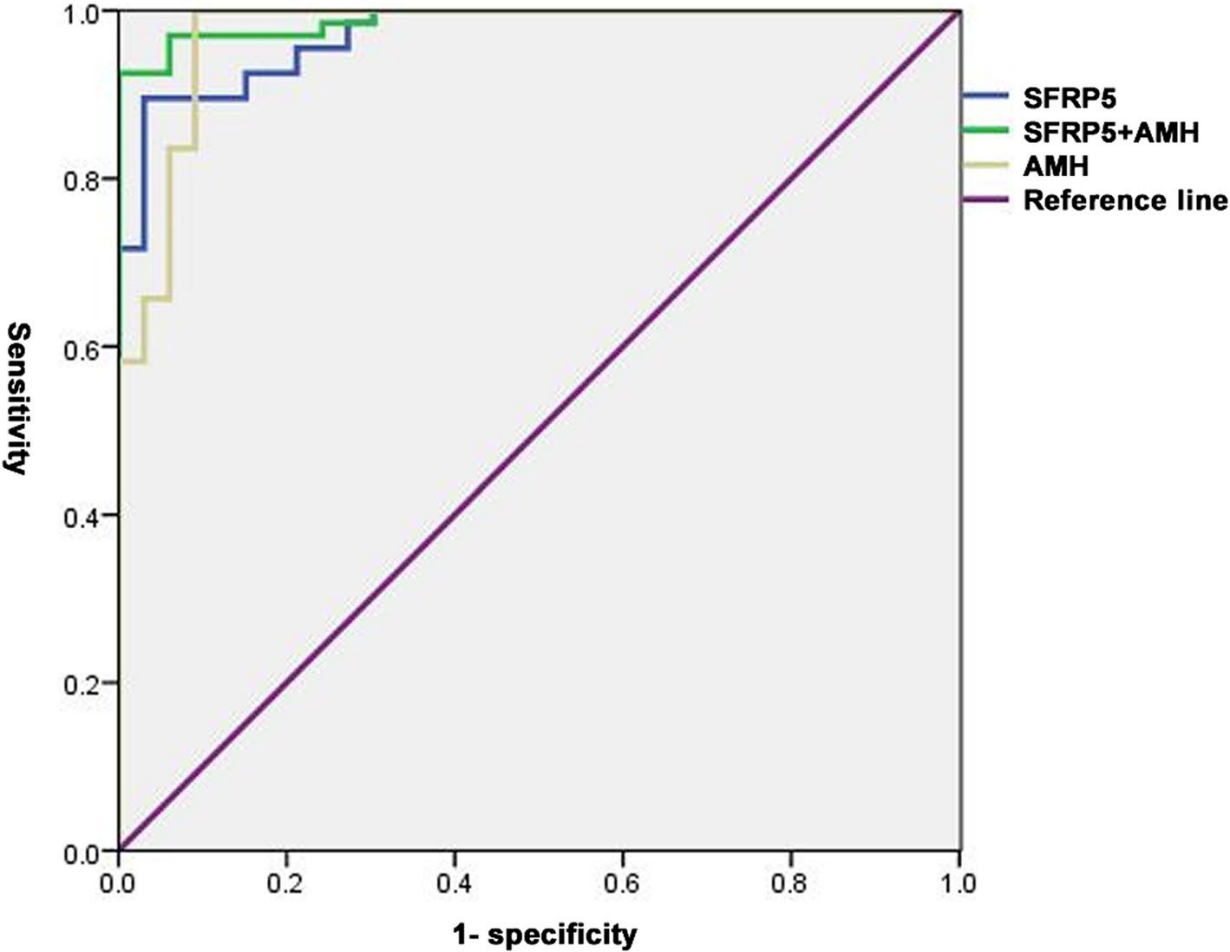
ROC curve analysis of SFRP5 for total PCOS. In all PCOS population, the SFRP5 cut-off value was 46.13 ng/ml (AUC 0.960; 95% CI 0.900–0.989; *P* < 0.0001) to identify PCOS with a sensitivity of 88.06% and specificity of 96.87%. The AMH cut-off value was 3.23 ng/ml (AUC 0.968; 95% CI 0.912–0.993; *P* < 0.0001) with a sensitivity of 98.51% and specificity of 90.62%. The AUC of combination of SFRP5 and AMH was 0.980 with a sensitivity of 91.04% and specificity of 100% (95% CI 0.930–0.998; *P* < 0.0001). AUC: area under the curve, ROC: receiver operating characteristic analysis

Multivariate logistic regression analysis indicated decreased plasma SFRP5 levels were associated with an increased incidence of PCOS (OR: 0.818, 95% confidence interval (CI):0.691–0.968, *P* = 0.019) (Table 4). The ROC analysis showed the cut-off point of SFRP5 was 46.13 ng/ml with a sensitivity of 88.06% and a higher specificity of 96.87% (AUC: 0.960, 95% CI: 0.900–0.989, *P* < 0.0001). The cut-off point of AMH was 3.23 ng/ml with a sensitivity of 98.51% and a specificity of 90.62% (AUC: 0.968, 95% CI 0.912–0.993, *P* < 0.0001). Combination of SFRP5 and AMH improved the specificity to 100% and still with a sensitivity of 91.04% (AUC: 0.980, 95% CI: 0.930–0.998, *P* < 0.0001) (Fig. 1).

**Table 4:** Multiple logistic regression analysis of SFRP5 for the diagnosis of [PCOS]

	OR	95% CI	*P-*value
Total PCOS	0.818	0.691–0.968	0.019
PCOS Subgroup (AMH < 4.7 ng/ml)	0.818	0.692–0.968	0.019

**Fig. 2:**
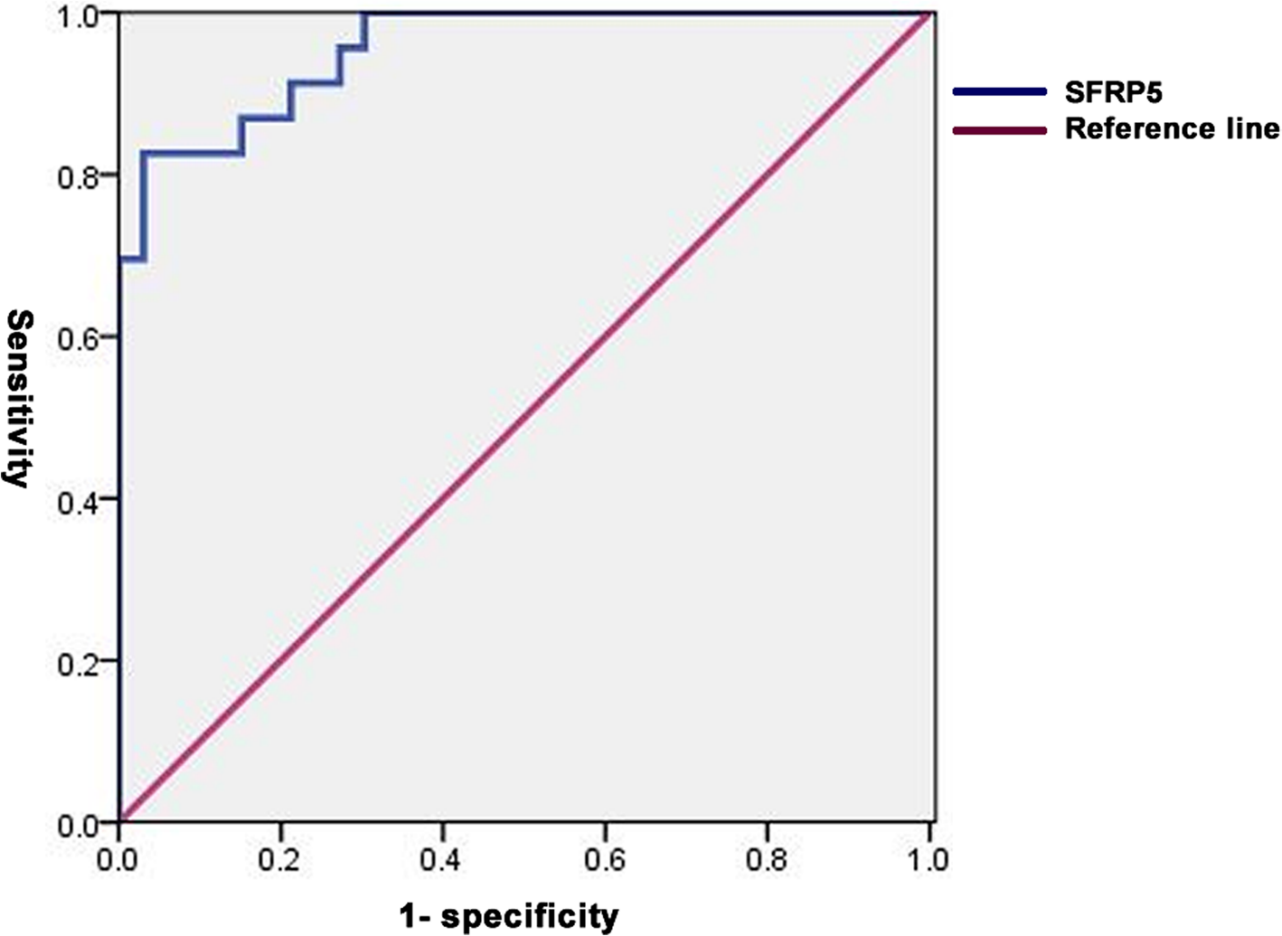
ROC curve analysis of SFRP5 for PCOS with AMH < 4.7 ng/ml. In PCOS with AMH < 4.7 ng/ml, the SFRP5 cut-off value was 42.69 ng/ml (AUC, 0.955; 95% CI 0.864–0.992; *P* < 0.0001) to identify PCOS with a sensitivity of 82.61% and specificity of 96.97%. AUC:area under the curve, ROC: receiver operating characteristic analysis

For women with a lower AMH level (< 4.7 ng/ml, N = 23), SFRP5 was still predictive of PCOS (OR: 0.818, 95% CI: 0.692–0.968, *P* = 0.019) (Table 4). The cut-off point of SFRP5 was 42.69 ng/ml with a higher specificity of 96.97% and a sensitivity of 82.61% (AUC: 0.955, 95% CI 0.864–0.992, *P* < 0.0001) (Fig. 2).

### Metformin promoted SFRP5 and decreased leptin, IL-6 and TNFα protein secretion in PCOS women with metabolic abnormalities

**Fig. 3:**
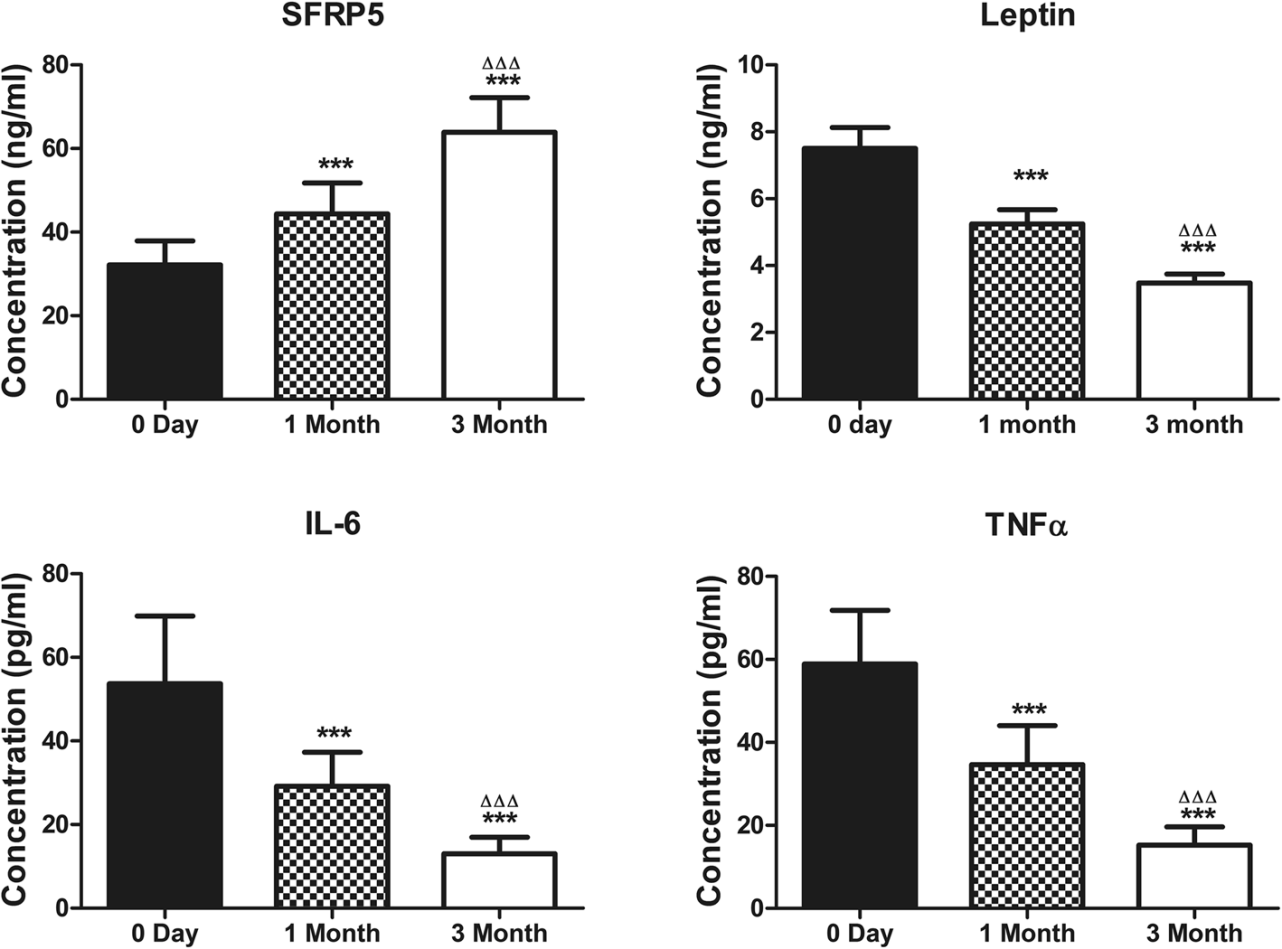
Comparison of the serum level of SFRP5, leptin, IL-6 and TNFα in PCOS women before and 1 month, 3 months after treatment with metformin in Group I PCOS women. *** compare with 0 Day, *P* < 0.001. ΔΔΔ compare with 1Month, *P* < 0.001

There were 73 PCOS women were included in the study (37 in Group I and 36 in Group II). There was no difference of baseline demographic and clinical characteristics between (Table 5). After treated with metformin for 1 and 3 months, serum SFRP5 of Group I significantly increased than that before administration (45.60 ± 8.148 and 63.92 ± 8.24 *vs* 32.16 ± 5.71 ng/ml, *P* < 0.001). Conversely, the secretion of leptin, IL-6 and TNFα were decreased after 1 and 3 months' treatment (*P* < 0.001) (Fig. 3).

**Table 5:** [Baseline] demographic and clinical characteristics between two treatment groups of clomiphene-metformin combination

Item	Group I (Clomiphene and Metformin)	Group II (Clomiphene alone)	*P*-value
N	37	36	
Age (years)	28.86 ± 0.53	27.36 ± 0.65	0.08
BMI (Kg/m^2^)	25.13 ± 0.36	25.08 ± 0.30	0.91
Duration of infertility (years)	2.17 ± 0.2277	1.911 ± 0.2116	0.42
Spontaneous menstrual cycle interval (months)	3.42 ± 0.38	2.66 ± 0.34	0.14
Menstrual flow length (days)	6.62 ± 0.55	6.53 ± 0.53	0.90
HbA1c (%)	5.79 ± 0.04	5.74 ± 0.05	0.53
HOMA-IR	3.30(2.56, 4.49)	3.42(2 .78, 3.91)	0.72

### Pretreatment with metformin had a positive effect on the ovulation rate and pregnancy rate of PCOS women with metabolic abnormalities

Before clomiphene treatment, the parameters of glucose metabolism in Group I were all adjusted to normal by metformin (data not shown). The ovulation rate, number of dominant follicles (*P* < 0.001), serum estradiol level on the day of HCG administration and accumulated clinical pregnancy rate (*P* < 0.05) in Group I was significantly higher than that of Group II. The duration for follicular development in Group I was significantly shorter than that of Group II (*P* < 0.01). There was no statistical significance in endometrial thickness, accumulated HCG positive rate and miscarriage rate between the two groups (Table 6).

**Table 6:** Comparison of the clinical outcomes between the two treatment groups of clomiphene-metformin combination

Item	Group I (Clomiphene and Metformin)	Group II (Clomiphene alone)	*P*-value
N	37	36	
Ovulation rate (%)	67.6(25)	28.6(8)	< 0.0001
days for follicular development	20.62 ± 1.34	26.69 ± 1.29	0.0017
Endometrial thickness (mm)	8.34 ± 0.34	8.15 ± 0.27	0.6663
Number of dominant follicles(> 14 mm)	1.76 ± 0.28	0.53 ± 0.20	0.0007
Serum estradiol on day of HCG (pg/mL)	564.6 ± 116.2	270.4 ± 62.0	0.0287
HCG positive rate (%)	37.8(14)	19.4(7)	0.1208
Clinical pregnancy rate (%)	29.7(11)	8.3(3)	0.0352
Miscarriage rate (%)	21.4(3)	57.1(4)	0.1564
Multiple pregnancy rate (%)	18.2(2)	33.3(1)	

## Discussion

As an anti-inflammatory adipokine, SFRP5 has been identified implicated in metabolic homeostasis. In this study, SFRP5 levels were negatively associated with incidence of PCOS, HA, IR and inflammation independent of obesity. Plasma SFRP5 level might have a potential role for the supplement of AMH in predicting PCOS. Metformin promoted SFRP5 protein secretion and decreased pro-inflammatory factors in PCOS women with improved ovulation rate and pregnancy rate.

Consistent with the findings of Hu, we found serum SFRP5 levels were decreased in PCOS in Chinese population [22], which was opposite to the findings of Almario in American population [21]. One difference was that we used Rotterdam criteria to include PCOS women, whereas Almario used the NIH criteria. The other difference was that the reference women reported by Almario were fatter (BMI: 29.0 ± 6.3 kg/m^2^) than our Chinese controls (BMI: 20(19.8, 21)). And they were older and with higher cholesterol level than their PCOS women. Because Chinese women have an increased risk of metabolic problems at a low BMI (≥ 24) [23], the control women included in our study have a relatively lower BMI. As BMI can not discriminate between fat mass and muscle mass and cannot represent abdominal adiposity, we detected WHR and found no relationship between SFRP5 and WHR. Consistent with the findings of Almario and other study [14], serum SFRP5 level was independent of obesity. Intensive exploration showed SFRP5 conditionally exerted an anti-inflammatory effect in response to an obesity-associated microenvironment [8, 9]. So the microenvironment rather than the mass of adipocytes plays a more important role in the regulation of SFRP5. In addition, the racial difference of SFRP5 should also be taken into accounted when SFRP5 is used as a specific biomarker for PCOS in the subsequent analysis.

We detected higher level of IL-6 and TNF-ɑ and FAI in PCOS, which were correlated with SFRP5. Inflammation is closely interrelated with HA in ovarian microenvironment. HA stimulated cumulus cells secreting more inflammatory cytokines as well as oocyte maturation. In turn, the direct exposure of ovarian theca cells to pro-inflammatory stimuli also increases androgen production [24]. Our findings suggest SFRP5 is involved in the interaction of inflammations and HA.

We demonstrated that FINS, 2-h INS, AUCI and HOMA-IR were significantly higher in PCOS, which indicated the association of IR and PCOS. Moreover, 2-h INS and 2-hBG were higher in normal-weight PCOS women than normal control indicating the metabolic aberration independent of obesity in PCOS. Serum SFRP5 was negatively correlated with FINS, 2-h INS, AUCI and HOMA-IR. The negative correlation between SFRP5 and IR has been determined in T2DM patients and animal models [25]. Caloric restriction improved insulin sensitivity and was associated with a significant increase of SFRP5. Proinflammatory cytokines and mediators closely correlate with insulin resistance through interfering with insulin signal transduction. Whether SFRP5 responds to hyperinsulinemia or the inflammatory conditions accompanied with IR need to be considered.

AMH, correlating with PCOM, has been proposed as a biomarker for the diagnosis of PCOS. However, AMH can be influenced by HA, obesity and inflammation. Moreover, the conflicting results of AMH’s effect on the growth of primordial follicles [26] and the heterogeneity of the PCOS phenotypes led to current controversy on the value of AMH for predicting PCOS among an unselected group of women. Our results suggest that SFRP5 could predict PCOS with a higher specificity in total PCOS population. However, the sensitivity of SFRP5 was lower than AMH. Combination of AMH and SFRP5 improved the sensitivity and specificity of the two biomarkers individually. In the subgroup of PCOS with lower AMH (< 4.7 ng/ml), SFRP5 still had a higher specificity. We proposed that SFRP5 worked as a specific biomarker for PCOS as complement to AMH especially in phenotype without PCOM.

The current therapeutic options for infertile PCOS women using clomiphene citrate (CC) et al. are always associated with drug resistance, especially in PCOS women with metabolic abnormalities. The effects of metformin on PCOS related IR, inflammation, ovarian steroidogenesis and ovulation have been confirmed. We found that metformin promoted SFRP5 protein secretion and decreased leptin, IL-6 and TNFα protein secretion of PCOS women in a time dependent manner. Meanwhile, our results identified that metformin combined with CC had positive effects on the ovulation rate and pregnancy rate of PCOS women with metabolic abnormalities. Accordingly, a similar conclusion was drawn in the 2017 Cochrane Review [27]. Our results suggest that SFRP5 related anti-inflammatory process is involved in the improvement of ovulation of PCOS by metformin.

As the heterogeneity of PCOS and small sample size of our study, the coefficient of variations (CVs) for some metabolic assays are relatively higher (Supplemental Table 1). This is similar to the other studies on SFRP5. And the differential expression of SFRP5 between Chinese and American suggests that a world-wide research with large sample size, different races and detailed grouping is needed to investigate the predictive value of SFRP5.

## Conclusions

SFRP5 was decreased in PCOS and associated with metabolic inflammation. SFRP5 has a potential role for the supplement of AMH in predicting PCOS. SFRP5 participates in the anti-inflammatory effect of metformin in improving follicular development. As this is a retrospective study with limited cases, a well-designed randomized controlled intervention trial is required to eliminate potential bias. The molecular mechanism of SFRP5's anti-inflammatory role in PCOS should be further investigated in vitro and in animal model.

## Supplementary Information

**Additional file 1: Supplemental Table 1**. Coefficient of variation for all metabolic assays.

## Data Availability

The data generated during the current study are available from the corresponding author on reasonable request.

## Abbreviations

PCOS: Polycystic Ovary Syndrome
SFRP: Secreted frizzled related protein
ROC: Receiver operating characteristic
HA: Hyperandrogenism
OA: Oligo-anovulation
PCOM: Polycystic ovarian morphology
IGT: Impaired glucose tolerance
IR: Insulin resistance
T2DM: Type 2 diabetes mellitus
BMI: Body mass index
HCG: Human chorionic gonadotropin
HbA1c: Hemoglobin A1c
HDL-C: High-density lipoprotein cholesterol
TG: Triglyceride
LDL-C: Low-density lipoprotein cholesterol
FSH: Follicle-stimulating hormone
LH: Luteinizing hormone
SHBG: Sex hormone-binding globulin
HOMA-IR: Insulin resistance index
FAI: Free androgen index
AUCG: Area under the curve of glucose
AUCI: Area under the curve of insulin
OGTT: Oral glucose tolerance test
ELISA: Enzyme-linked immunosorbent assay
SD: Standard deviation
WHR: Waist hip ratio
