# Correction to: Decreased SFRP5 correlated with excessive metabolic inflammation in polycystic ovary syndrome could be reversed by metformin: implication of its role in dysregulated metabolism

**DOI:** 10.1186/s13048-021-00855-4

**Published:** 2021-08-12

**Authors:** Yi Zhang, Yuxin Ran, Lingna Kong, Lihong Geng, Hua Huang, Hongying Zhang, Jun Hu, Hongbo Qi, Ying Chen

**Affiliations:** 1 NHC Key Laboratory of Birth Defects and Reproductive Health, Chongqing Population and Family Planning Science and Technology Research Institute, Chongqing, People’s Republic of China; 2 Reproductive Medicine Center, Department of Obstetrics and Gynecology, The First Affiliated Hospital of Chongqing Medical University, Chongqing, People’s Republic of China; 3 School of Nursing, The First Affiliated Hospital of Chongqing Medical University, Chongqing, People’s Republic of China

**Correction to: J Ovarian Res 14, 97 (2021)**

**https://doi.org/10.1186/s13048-021-00847-4**

In the original publication of this article [1], the legend for Figs. 1 and 2 were missing. The correct figures are shown here. The original article has been corrected.

**Fig. 1:**
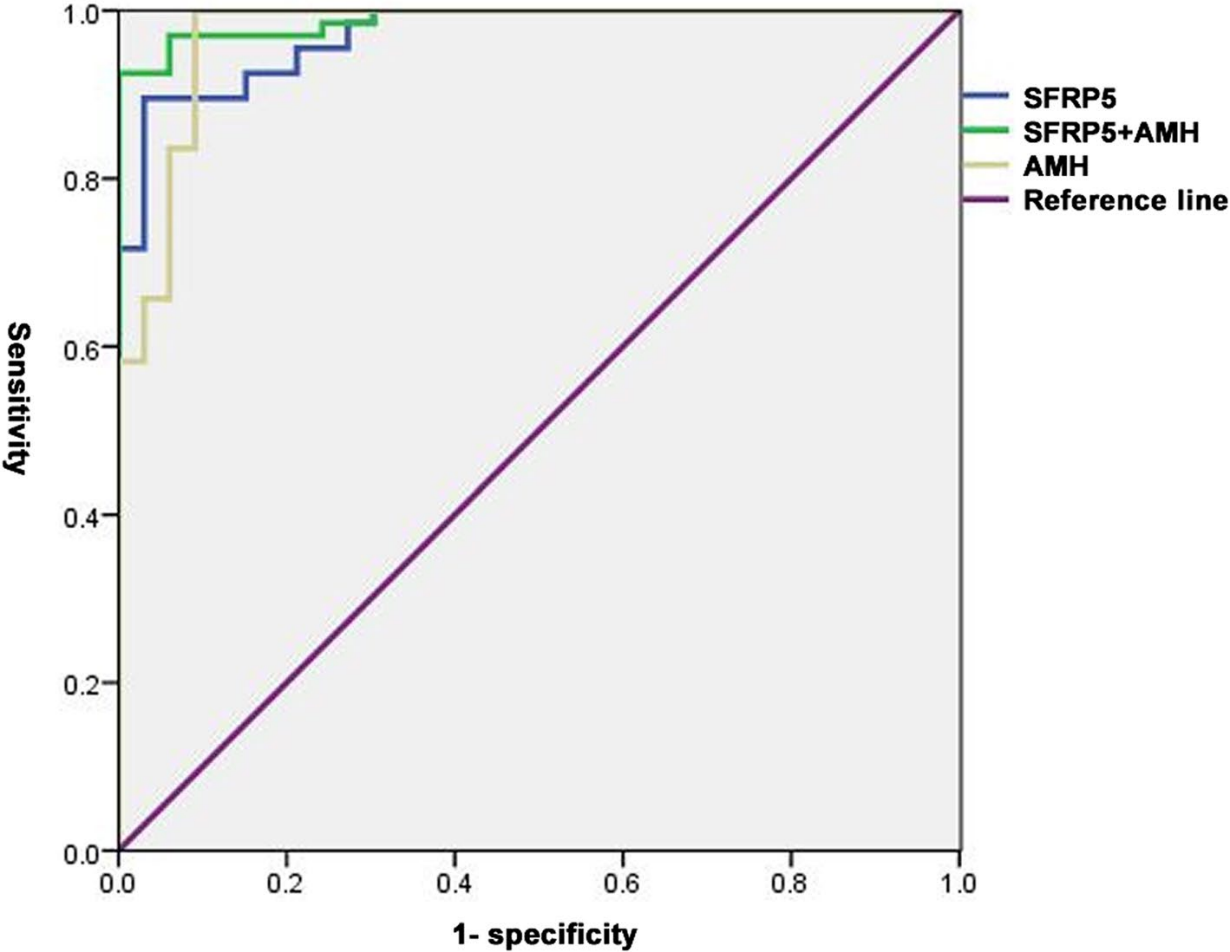
ROC curve analysis of SFRP5 for total PCOS. In all PCOS population, the SFRP5 cut-off value was 46.13 ng/ml (AUC 0.960; 95% CI 0.900–0.989; *P* < 0.0001) to identify PCOS with a sensitivity of 88.06% and specificity of 96.87%. The AMH cut-off value was 3.23 ng/ml (AUC 0.968; 95% CI 0.912–0.993; *P* < 0.0001) with a sensitivity of 98.51% and specificity of 90.62%. The AUC of combination of SFRP5 and AMH was 0.980 with a sensitivity of 91.04% and specificity of 100% (95% CI 0.930–0.998; *P* < 0.0001). AUC: area under the curve, ROC: receiver operating characteristic analysis

**Fig. 2:**
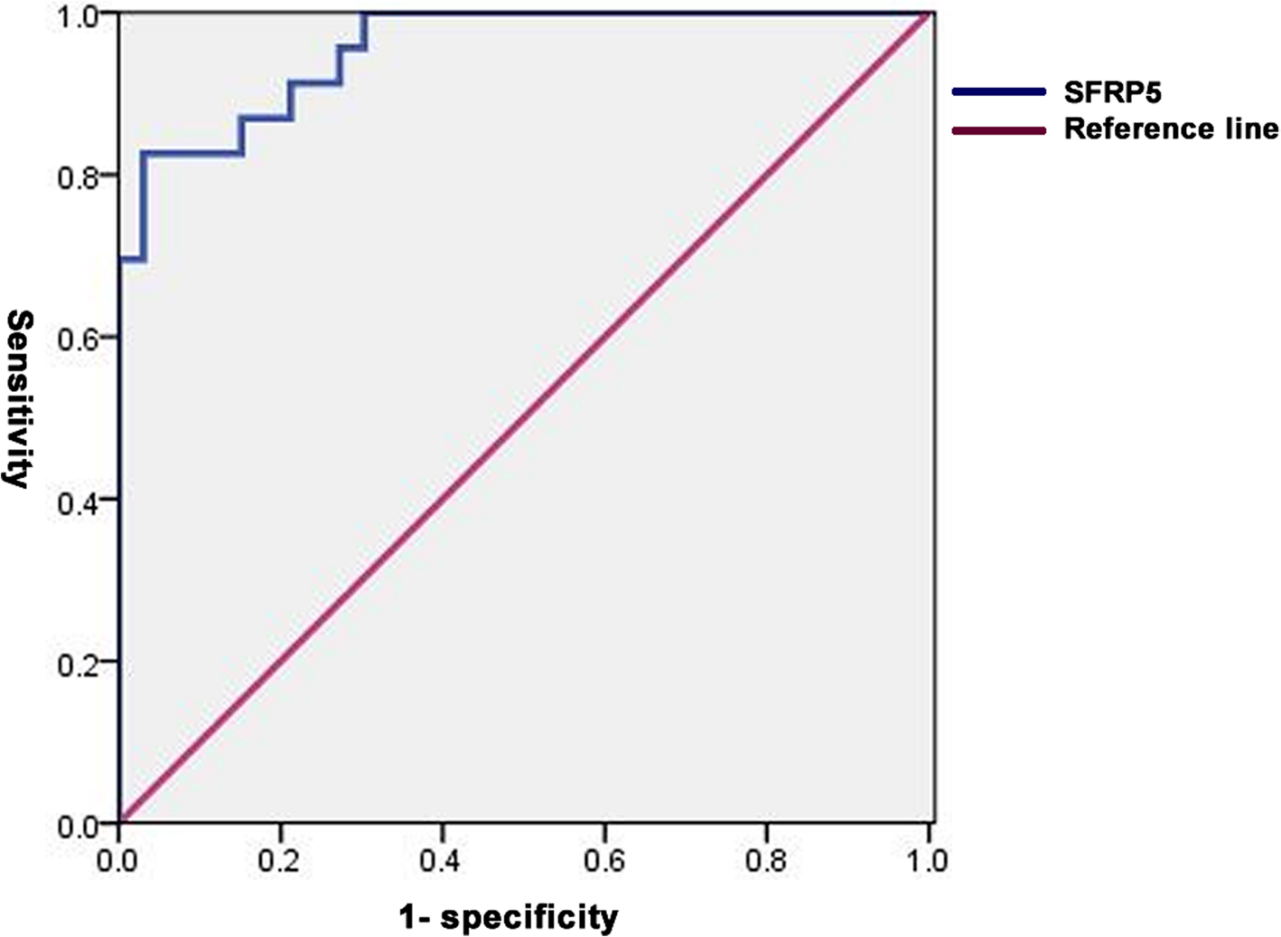
ROC curve analysis of SFRP5 for PCOS with AMH < 4.7 ng/ml. In PCOS with AMH < 4.7 ng/ml, the SFRP5 cut-off value was 42.69 ng/ml (AUC, 0.955; 95% CI 0.864–0.992; *P* < 0.0001) to identify PCOS with a sensitivity of 82.61% and specificity of 96.97%. AUC: area under the curve, ROC: receiver operating characteristic analysis
