# Supplementary material for: Decreased SFRP5 correlated with excessive metabolic inflammation in polycystic ovary syndrome could be reversed by metformin: implication of its role in dysregulated metabolism
Source: J Ovarian Res. 2021 Jul 20;14:97. doi: 10.1186/s13048-021-00847-4 (PMC8293500; doi:10.1186/s13048-021-00847-4)
Supplement: Supplementary file 1 — Additional file 1: Supplemental Table 1. Coefficient of variation for all metabolic assays. [file 13048_2021_847_MOESM1_ESM.docx]

**Supplemental Table 1 Coefficient of variation** **for all metabolic assays**

| **Item** |  | **Coefficient of variation (%)** | |
| --- | --- | --- | --- |
|  | **Normal control** | **PCOS** | |
|  |  | **Normal weight**  **(BMI<25)** | **Overweight /Obese**  **(BMI≥25)** |
| **N** | **33** | **33** | **34** |
| **SFRP5(ng/ml)** | 19.4 | 28.4 | 25.4 |
| **AMH(ng/ml)** | 24.6 | 36.5 | 42.8 |
| **FSH(mIU/ml)** | 25.8 | 30.2 | 32.0 |
| **LH(mIU/ml)** | 25.2 | 80.1 | 66.3 |
| **FAI** | 26.2 | 41.3 | 44.6 |
| **FINS(Uiu/ml)** | 35.8 | 59.9 | 36.4 |
| **FBG(mmol/l)** | 4.7 | 6.3 | 10.7 |
| **TG(mmol/L)** | 22.5 | 49.5 | 40.3 |
| **TC(mmol/L)** | 10.1 | 11.4 | 11.1 |
| **HDL-C(mmol/L)** | 21.7 | 21.1 | 18.0 |
| **LDL-C(mmol/L)** | 15.6 | 13.3 | 14.0 |
| **HbA1c(%)** | 4.5 | 5.0 | 4.8 |
| **HOMA-IR** | 30.8 | 63.9 | 38.3 |
| **IL6(pg/ml)** | 65.6 | 64.4 | 74.7 |
| **TNFα(ng/ml)** | 19.7 | 22.3 | 17.9 |
